# Impact of adjuvant therapy after radical hysterectomy for early-stage cervical cancer on mortality and recurrence: a systematic review and meta-analysis

**DOI:** 10.3389/fonc.2026.1743116

**Published:** 2026-04-29

**Authors:** Ying Wang, Zhen-Ni Yue, Hua Yang

**Affiliations:** Department of Gynecology, The Second Affiliated Hospital of Guilin Medical University, Guilin, Guangxi, China

**Keywords:** adjuvant therapy, cervical cancer, chemoradiotherapy, meta-analysis, mortality, radical hysterectomy, radiotherapy, recurrence

## Abstract

**Background:**

The role of adjuvant therapy following radical hysterectomy in early-stage cervical cancer remains controversial. Although adjuvant radiotherapy or chemoradiotherapy is frequently administered to reduce recurrence in patients with adverse pathological features, its effect on overall survival and recurrence prevention remains uncertain. This systematic review and meta-analysis aimed to evaluate the impact of adjuvant therapy after radical hysterectomy on recurrence and mortality in patients with early-stage cervical cancer.

**Methods:**

This study followed the Preferred Reporting Items for Systematic Reviews and Meta-Analyses (PRISMA) guidelines. Comprehensive searches of PubMed, Embase, Web of Science, and the Cochrane Library were performed from inception to October 1, 2025. Eligible studies included randomized controlled trials and observational cohorts comparing radical hysterectomy alone with radical hysterectomy plus adjuvant therapy in patients with FIGO stage IA–IIA cervical cancer. Pooled risk ratios (RRs) with 95% confidence intervals (CIs) were calculated using fixed- or random-effects models according to heterogeneity. Publication bias and sensitivity analyses were conducted to ensure result robustness.

**Results:**

Eight studies comprising 1, 396 patients were included. Compared with surgery alone, adjuvant therapy did not significantly reduce recurrence risk (RR = 1.51, 95% CI: 0.75–2.28, I² = 76.2%, p < 0.001) or overall mortality (RR = 1.40, 95% CI: 0.55–2.25, I² = 0.0%, p = 0.519). Subgroup analyses stratified by study design, FIGO stage, and adjuvant regimen confirmed the consistency of findings, and sensitivity analyses demonstrated stability of the pooled estimates. No evidence of publication bias was detected.

**Conclusions:**

Adjuvant therapy after radical hysterectomy did not significantly improve recurrence or mortality outcomes in early-stage cervical cancer. These findings support a selective, risk-adapted approach to postoperative management, minimizing unnecessary treatment-related toxicity and optimizing individualized care.

**Systematic review registration:**

https://www.crd.york.ac.uk/prospero/, identifier CRD420251265823.

## 1 Introduction

Cervical cancer remains one of the most prevalent malignancies among women worldwide, ranking as the fourth most common cancer in both incidence and mortality, despite significant advances in screening and vaccination strategies (1). According to recent global cancer statistics, approximately 660, 000 new cases and 350, 000 deaths were reported in 2023, with the majority occurring in low- and middle-income countries where access to early detection and comprehensive treatment remains limited (2, 3). Early-stage cervical cancer, defined as stages IA to IIA according to the International Federation of Gynecology and Obstetrics (FIGO) classification, is typically managed with radical hysterectomy and pelvic lymphadenectomy (4). This surgical approach achieves excellent local control and long-term survival rates exceeding 85% in appropriately selected patients. However, postoperative management strategies for patients with intermediate- or high-risk pathological features remain a matter of clinical controversy (5–7).

Adjuvant therapy following radical hysterectomy, including radiotherapy, chemotherapy, or concurrent chemoradiotherapy, is commonly recommended to reduce the risk of recurrence in patients with adverse prognostic factors such as deep stromal invasion, lymphovascular space invasion (LVSI), large tumor size, or positive surgical margins (8). These pathological characteristics are known to correlate with an increased risk of locoregional and distant recurrence. The rationale for adjuvant treatment lies in eradicating potential microscopic residual disease and improving overall survival outcomes (9). Nevertheless, the actual benefit of adjuvant therapy in patients with early-stage cervical cancer remains uncertain (10, 11). While previous randomized controlled trials and cohort studies have demonstrated survival benefits of chemoradiotherapy in patients with high-risk features (e.g., positive nodes or parametrial invasion), its role in intermediate-risk or low-risk patients is less clear. The use of postoperative adjuvant therapy also introduces substantial treatment-related toxicity, including bowel and bladder dysfunction, vaginal stenosis, and impaired quality of life, which must be weighed against potential survival gains (12, 13). Recent clinical trials and meta-analyses have attempted to define optimal adjuvant strategies in early-stage cervical cancer, yet results have been inconsistent. Some studies have suggested that adjuvant chemoradiotherapy confers modest improvements in recurrence-free survival, while others report no statistically significant advantage over surgery alone. Furthermore, the heterogeneity in patient selection criteria, adjuvant regimens, radiation doses, and follow-up durations across published studies complicates the interpretation of results (11, 13, 14). The Sedlis criteria, established from earlier clinical trials, remain widely referenced to guide adjuvant therapy decisions; however, the applicability of these criteria to contemporary practice has been questioned due to evolving surgical techniques, improved staging accuracy, and the introduction of minimally invasive approaches. Thus, the lack of consensus has contributed to variation in clinical practice and ongoing uncertainty regarding which subgroups truly benefit from postoperative adjuvant therapy (1, 15).

Given these controversies, a comprehensive reassessment of available evidence is warranted. Systematic evaluation and meta-analysis of published data can provide a more precise estimation of the oncologic benefit of adjuvant therapy after radical hysterectomy for early-stage cervical cancer, particularly with respect to recurrence and mortality outcomes. Therefore, this systematic review and meta-analysis aimed to evaluate the impact of adjuvant therapy following radical hysterectomy on recurrence and mortality in patients with early-stage cervical cancer, integrating recent clinical evidence to inform evidence-based recommendations for postoperative management.

## 2 Methods

### 2.1 Search strategy

This systematic review and meta-analysis was conducted following the Preferred Reporting Items for Systematic Reviews and Meta-Analyses (PRISMA) guidelines (16). A comprehensive literature search was performed across PubMed, Embase, Web of Science, and The Cochrane Library to identify studies evaluating the effects of adjuvant therapy after radical hysterectomy for early-stage cervical cancer on mortality and recurrence. The search covered all publications from database inception to October 1, 2025, without language restrictions, and non-English articles with English abstracts were included to ensure completeness. Both Medical Subject Headings (MeSH) terms and free-text keywords were used in combination to enhance search sensitivity and specificity, with search concepts focusing on cervical cancer, radical hysterectomy, adjuvant therapy, mortality, and recurrence. In addition to database searches, reference lists of included studies and relevant reviews were manually screened to identify additional eligible articles, and other potential sources were consulted to supplement the literature. Detailed search strategies for each database are provided in **Supplementary Table 1**. The protocol for this systematic review was registered in PROSPERO (CRD420251265823).

### 2.2 Inclusion criteria and exclusion criteria

Studies were included if they met the following criteria:

1. The study population consisted of patients with early-stage cervical cancer, defined according to the FIGO staging system (stages IA–IIA).
2. Patients underwent radical hysterectomy with or without lymph node dissection as the primary surgical treatment.
3. The intervention group received any form of adjuvant therapy, including adjuvant radiotherapy, chemotherapy, or concurrent chemoradiotherapy, following radical hysterectomy, while the control group did not receive postoperative adjuvant treatment.
4. The study reported at least one of the following outcome measures: overall survival (OS), disease-free survival (DFS), recurrence rate, or mortality rate.
5. The study design was a randomized controlled trial (RCT), prospective cohort study, or retrospective cohort study.

Studies were excluded if they met any of the following criteria:

1. The population included patients with advanced-stage or recurrent cervical cancer, or those who did not undergo radical hysterectomy.
2. The intervention was non-surgical or did not involve adjuvant therapy following radical hysterectomy.
3. The study lacked a control group for comparison.
4. The reported data were incomplete, duplicated, or could not be extracted or synthesized.
5. The article was a review, meta-analysis, case report, editorial, letter, or conference abstract without sufficient primary data.

### 2.3 Literature screening and data extraction

Data were independently extracted by two reviewers using a standardized form, and discrepancies were resolved through discussion or consultation with a third investigator. Extracted information included the first author and publication year, study design, study period, total number of patients and numbers in each group (radical hysterectomy alone vs. radical hysterectomy plus adjuvant radiotherapy), median age and age range, FIGO stage, median follow-up duration, histological type and grade, median tumor size, presence of lymphovascular invasion (LVI), and depth of stromal infiltration. When available, outcome data such as overall survival, disease-free survival, recurrence rate, and mortality rate were also collected.

### 2.4 Quality assessment

The quality of the included studies was independently assessed by two reviewers, and the results were cross-checked to ensure consistency. Any disagreements were resolved through discussion or consultation with a third reviewer. Randomized controlled trials were evaluated using the Cochrane Risk of Bias Assessment Tool (RoB 2.0) (17), which assesses bias across domains such as randomization process, deviations from intended interventions, missing outcome data, measurement of the outcome, and selection of the reported result. For cohort studies, the Newcastle–Ottawa Scale (NOS) (13) was used to evaluate study quality based on three domains: selection of study participants, comparability between study groups, and ascertainment of outcomes. The NOS has a maximum score of nine points, with higher scores indicating higher methodological quality (18).

### 2.5 Statistical analyses

All statistical analyses were performed using Stata version 18.0 (StataCorp LLC, College Station, TX, USA) and Review Manager (RevMan) version 5.4 (Cochrane Collaboration, Oxford, UK). Pooled effect estimates were calculated using hazard ratios (HRs), odds ratios (ORs), or risk ratios (RRs) with corresponding 95% confidence intervals (CIs), depending on the data reported in the included studies. When HRs or their 95% CIs were not directly available, they were estimated from Kaplan–Meier survival curves or other statistical information using established methods. Between-study heterogeneity was assessed using the Cochrane Q test and quantified by the I² statistic. A fixed-effect model (Mantel–Haenszel method) was applied when heterogeneity was low (I² ≤ 50% and P ≥ 0.10), whereas a random-effects model (DerSimonian–Laird method) was used when significant heterogeneity was detected (I² > 50% or P < 0.10). Sensitivity analyses were performed by sequentially excluding individual studies to evaluate the robustness of the pooled results. Publication bias was assessed using Begg’s rank correlation test and Egger’s regression asymmetry test. When significant publication bias was observed (P < 0.05), the trim-and-fill method was applied to estimate the potential impact of missing studies on the overall results. A P-value < 0.05 was considered statistically significant for all analyses.

## 3 Results

### 3.1 Search results and study selection

**Figure 1:**
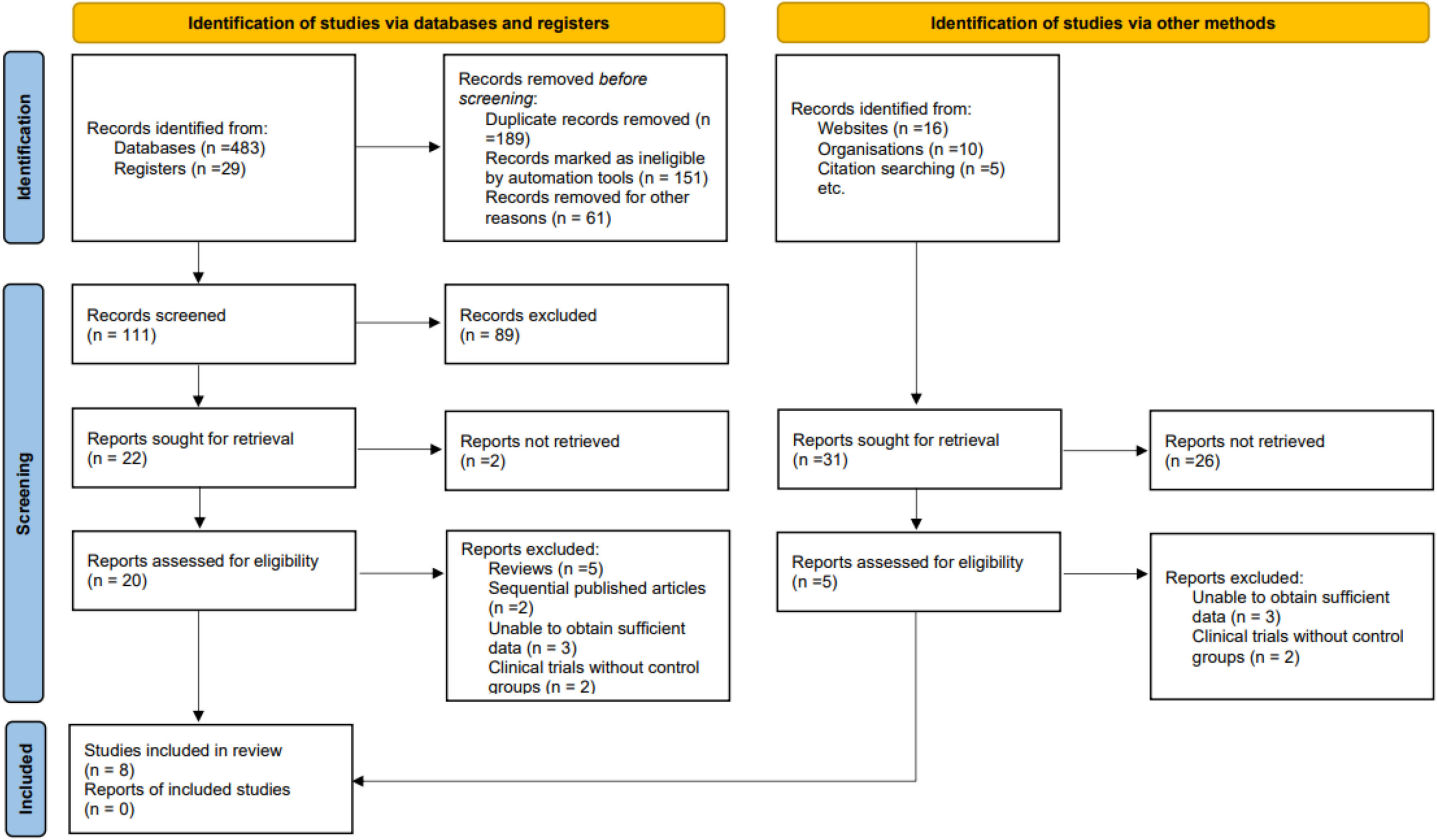
PRISMA flow diagram illustrating the process of literature identification, screening, eligibility assessment, and inclusion of studies in the final meta-analysis.

A total of 512 records were initially identified through database and registry searches, including 483 from electronic databases and 29 from trial registers. After removal of duplicates and records excluded prior to screening, 111 records underwent title and abstract screening, of which 89 were excluded. Twenty-two full-text articles were sought for retrieval, and 20 were successfully retrieved and assessed for eligibility. Of these, 12 were excluded due to being reviews, sequential publications, having insufficient extractable data, or lacking an appropriate control group, leaving 8 studies eligible for inclusion in the quantitative synthesis. Additional records identified through other sources (websites, organisations, and citation searching) did not yield any eligible studies after full-text assessment. Overall, 8 studies were included in the final review, with no multiple reports of included studies (19–26). The study selection process is summarized in **Figure 1**.

### 3.2 Study characteristics

A total of eight studies were included, involving 1, 396 patients with early-stage cervical cancer who underwent radical hysterectomy. Among them, 842 patients (60.3%) received surgery alone (SG group), and 554 patients (39.7%) received surgery combined with adjuvant radiotherapy (SG + RT group). The included studies consisted of two randomized controlled trials, two prospective cohort studies, and four retrospective studies. The median age of patients ranged from 20 to 88 years, and most cases were classified as FIGO stages IA–IIA. The median follow-up duration varied from 24 to 249 months. Squamous cell carcinoma was the predominant histological type (66.3%), followed by adenocarcinoma and adenosquamous carcinoma (22.1%). Overall, 35.2% of patients showed lymphovascular invasion, and 27.5% had deep stromal infiltration. The included evidence was derived predominantly from lymph node-negative or intermediate-risk postoperative populations. Studies specifically focused on node-positive disease were not adequately represented in the final quantitative synthesis. After reassessment of pathological risk composition, 7 of the 8 included studies (1, 276/1, 396 patients, 91.4%) predominantly enrolled node-negative or intermediate-risk early-stage populations. No included study was primarily composed of uniformly defined high-risk patients with positive nodes, positive margins, or parametrial invasion. One older randomized study (120/1, 396 patients, 8.6%) did not provide sufficiently detailed pathological stratification; however, it did not contribute extractable recurrence or mortality event counts to the main event-based pooled analyses. Detailed baseline characteristics and outcome data, including recurrence and mortality events, are summarized in **Table 1**.

**Table 1:** Baseline and pathological characteristics of included studies.

Author	Year	Study design	Patients (n)	SG (n)	SG + RT (n)	Median follow-up (months)	Median age (years, range)	FIGO Stage	Median tumor size (mm)	Histological type	Histological grade	LVI	Stromal Infiltration	Adjuvant group (n/N) – recurrence	Surgery-only (n/N) – recurrence	Adjuvant group (n/N) – deaths	Surgery-only (n/N) – deaths
Cibula et al. (22)	2018	Retrospective	231	127	104	10 years	All: 47.1 ± 13.1; SG: 47.0 ± 13.5; RT: 47.3 ± 12.5	IB	SG: <20 34, 20–40 78, >40 15; RT: <20 21, 20–40 61, >40 21	SG: SCC 96, AC 27, AS 4; RT: SCC 67, AC 29, AS 8	SG: G1 10, G2 59, G3 55; RT: G1 13, G2 41, G3 49	SG: 67; RT: 78 (p = 0.001)	SG: 13.0; RT: 13.4	12/104	8/127	15/104	9/127
Nakamura et al. (21)	2016	Retrospective	75	46	29	84.3 (47–135)	SG: 46.8 (27–78); RT: 44.7 (27–67); RT + BT: 49.9 (31–69); CRT: 63.5 (55–72); CRT + BT: 38 (33–43); CT: 53 (34–66)	IB1	<20 6; 20–40 69	SCC 51, AC 20, AS 4	NA	32	<1/2 29; >1/2 46	1/29	0/46	1/29	0/46
Pieterse et al. (24)	2006	Retrospective	51	17	34	60 (0–223)	SG: 42; RT: 44	SG: IB 12, IIA 5; RT: IB 28, IIA 6	SG: <40 4, ≥40 13; RT: <40 7, ≥40 27	SG: SCC 15, AC 1, AS 1; RT: SCC 28, AC 5, AS 1	NA	SG: 13; RT: 26	SG: ≤15 4, >15 11; RT: ≤15 5, >15 29	4/34	7/17	NR	NR
Rushdan et al. (20)	2004	Prospective	238	180	58	SG: 61.6 (1–103); RT: 74.3	NA	IB2: 35	21	SCC 64, AC 28, AS 3	G1 31, G2 42, G3 22	7	DSI: 7	2/27	0/28	NR	NR
Sedlis et al. (19)	1999	RCT	277	140	137	10 years (0.003–16)	41 (20–80)	IB	<2 cm 38; 2–4 cm 157; >4 cm 74	SCC 218, AC 27, AS 32	NA	195	>1/3 275; <1/3 2	21/137	39/140	18/137	30/140
Snijders et al. (25)	1999	Prospective	233	156	77	39 (2–128)	SG: 39; RT: 43	SG: IA 8, IB 135, IIA 12; RT: IA 1, IB 63, IIA 13	SG: <40 150, >40 5; RT: <40 57, >40 20	SG: SCC 108, AC 40, AS 8; RT: SCC 60, AC 11, AS 6	NA	10	SG: <15 148, >15 5; RT: <15 54, >15 21	23/77	12/156	NR	NR
Schorge et al. (23)	1997	Retrospective	171	116	55	84 (27–249)	46 (24–87)	IB1: SG 104, RT 31; IB2: SG 5, RT 16; IIA: SG 7, RT 8	SG: <20 78, >20 36; RT: <20 17, >20 37	SG: SCC 81, Non-SCC 35; RT: SCC 43, Non-SCC 12	SG: G1–2 85, G3 27; RT: G1–2 38, G3 14	SG: 46; RT: 31	SG: <2/3 85, >2/3 29; RT: <2/3 11, >2/3 42	11/55	17/116	NR	NR
Bilek et al.(26)	1982	RCT	120	60	60	44 (24–72)	All: 40.6 (23–60); SG: 42 (23–59); RT: 39 (23–60)	IB	NA	SCC 120	NA	NA	NA	NR	NR	NR	NR

NA, not available; NR, not reported; SG, surgery group; RT, radiotherapy plus surgery group; FIGO, International Federation of Gynecology and Obstetrics; LVI, lymphovascular invasion;

DSI, deep stromal infiltration; SCC, squamous cell carcinoma; AC, adenocarcinoma;

AS, adenosquamous carcinoma; RH, radical hysterectomy; RCT, randomized controlled trial;

BT, brachytherapy; CRT, chemoradiotherapy; CT, chemotherapy; FU, follow-up.

### 3.3 Results of quality assessment

**Figure 2:**
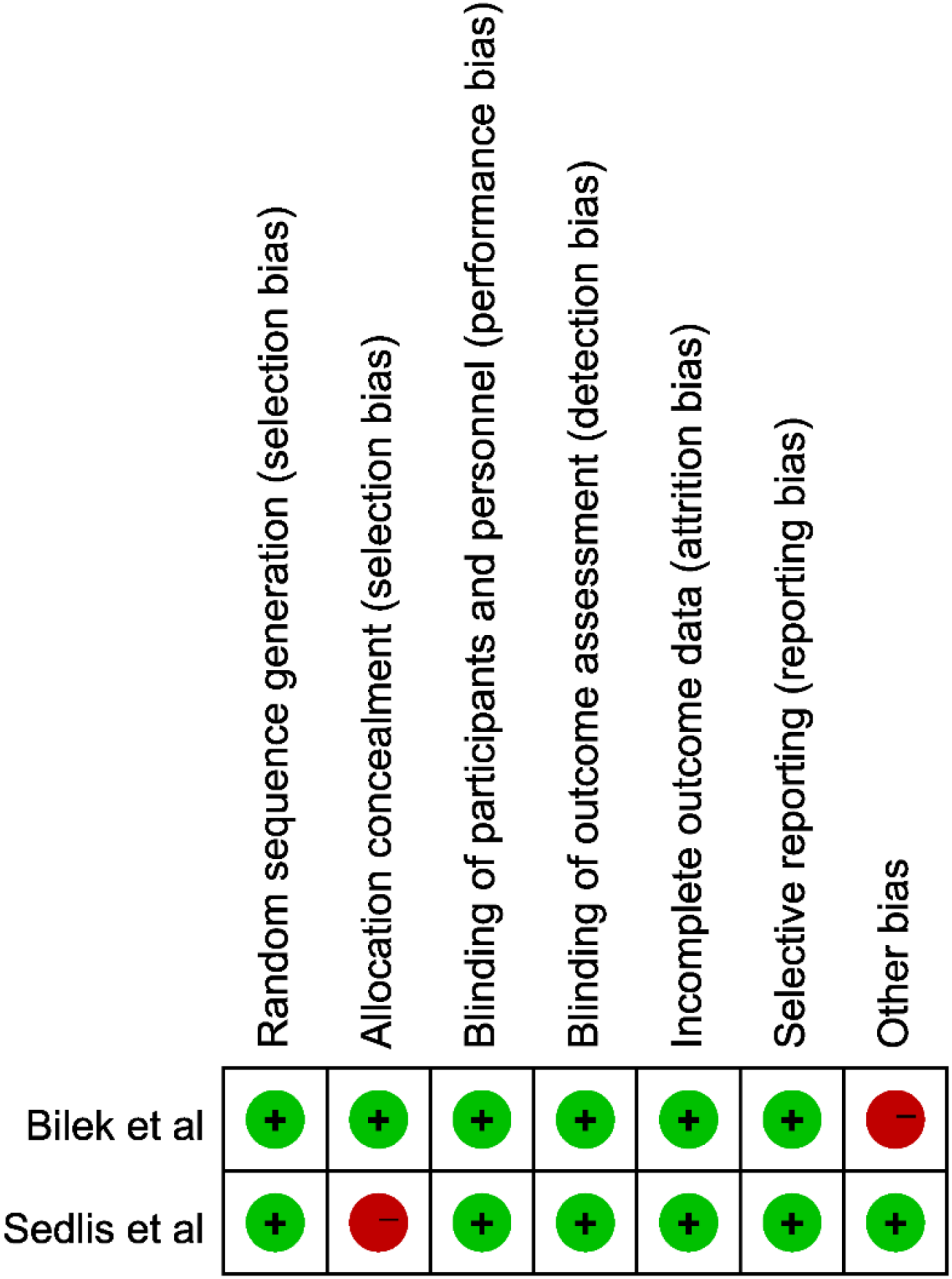
Summary of the quality assessment of included studies using the Cochrane RoB 2.0 tool for randomized controlled trials and the Newcastle–Ottawa Scale (NOS) for cohort studies.

The methodological quality of the included studies was generally acceptable. Randomized controlled trials were evaluated using the RoB 2.0 tool, which indicated an overall low risk of bias across most domains, including random sequence generation, blinding, and outcome assessment. Minor concerns were noted in allocation concealment and other bias domains (**Figure 2**). For the observational studies, quality was assessed using the NOS. The total NOS scores ranged from 7 to 9, indicating moderate to high methodological quality. Most cohort studies demonstrated good representativeness of the exposed and non-exposed groups, clear ascertainment of exposure, adequate comparability between cohorts, and sufficient duration and completeness of follow-up (**Table 2**). Overall, both randomized and non-randomized studies exhibited satisfactory methodological rigor, supporting the reliability of the pooled outcomes in this meta-analysis.

**Table 2:** The quality assessment according to Newcastle-Ottawa Scale of each cohort study.

Study	Representativeness of the exposed cohort	Selection of the non-exposed cohort	Ascertainment of exposure	Demonstration that outcome of interest was not present at start of study	Comparability of cohorts on the basis of the design or analysis	Assessment of outcome	Was follow-up long enough	Adequacy of follow up of cohorts	Total score
Cibula et al.	1	1		1	1	1	1	1	7
Nakamura et al.	1		1	1	1	1	1	1	7
Pieterse et al.	1	1	1	1	1	1	1	1	8
Ruhsdan et al.	1	1	1	1	2	1	1	1	9
Snijders et al.	1	1	1	1	2	1		1	8
Schoorge et al.	1	1	1	1	2	1	1	1	9

### 3.4 Recurrence outcomes

**Figure 3:**
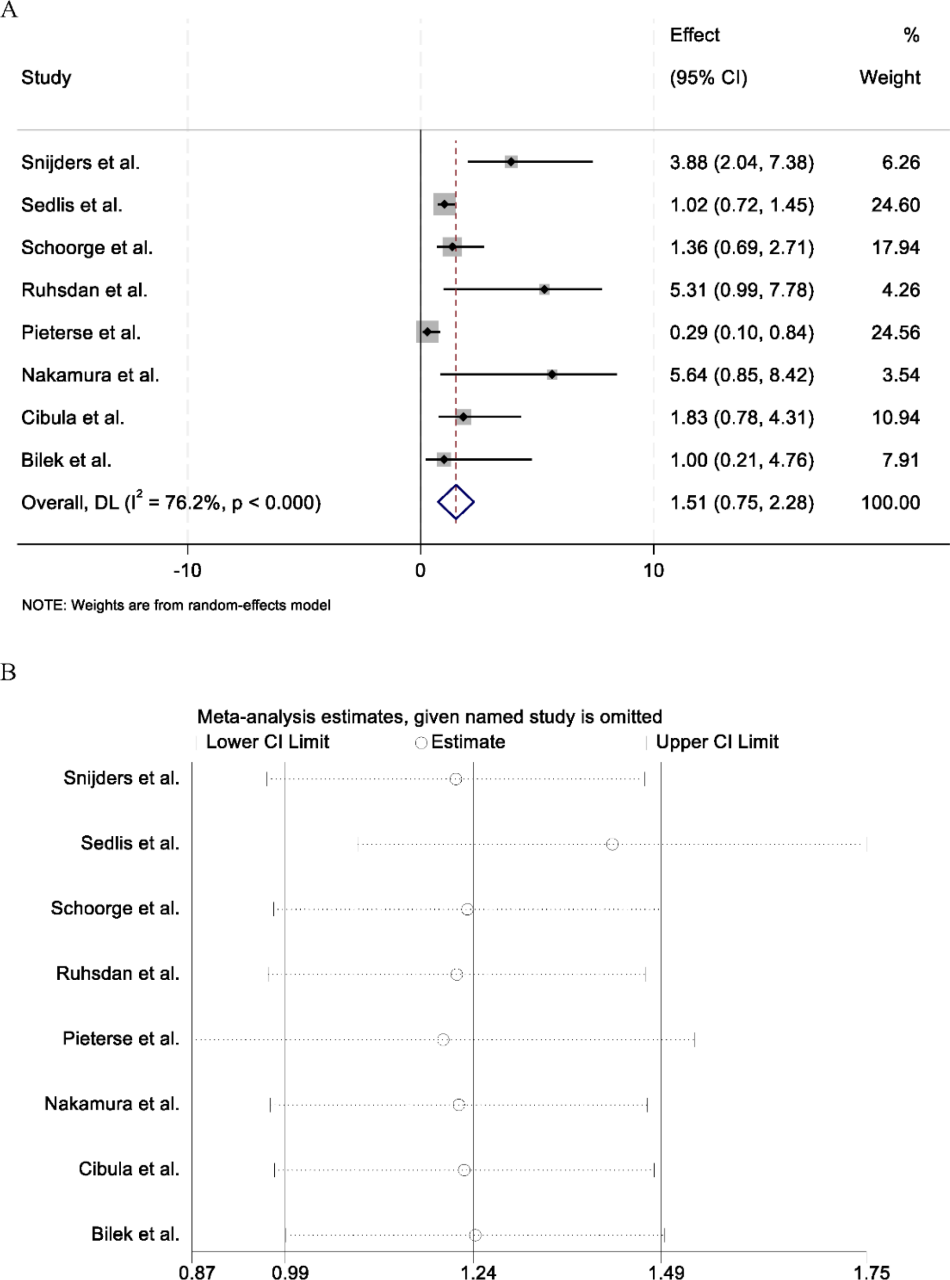
Forest plot **(A)** showing the pooled analysis of recurrence outcomes comparing surgery alone (SG) with surgery plus adjuvant therapy (SG + RT) in patients with early-stage cervical cancer. Effect sizes are expressed as risk ratios (RRs) with 95% confidence intervals (CIs), and the horizontal axis represents the logarithmic scale of the risk ratio. **(B)** Sensitivity analysis demonstrating the stability of the pooled estimates after sequential exclusion of individual studies.

A total of eight studies reported data on tumor recurrence among patients who underwent radical hysterectomy for early-stage cervical cancer. Considerable heterogeneity was observed across the included studies (I² = 76.2%, p < 0.001); therefore, a random-effects model was applied for the pooled analysis. The combined results demonstrated that there was no statistically significant difference in recurrence rates between patients who received adjuvant therapy after surgery and those who underwent surgery alone (RR = 1.51, 95% CI: 0.75–2.28) (**Figure 3A**). Sensitivity analysis was performed by sequentially omitting individual studies to assess the robustness of the pooled estimate. The overall effect size and heterogeneity remained stable, indicating that no single study disproportionately influenced the results (**Figure 3B**). These findings suggest that adjuvant therapy after radical hysterectomy may not significantly reduce the risk of recurrence in patients with early-stage cervical cancer.

### 3.5 Mortality outcomes

**Figure 4:**
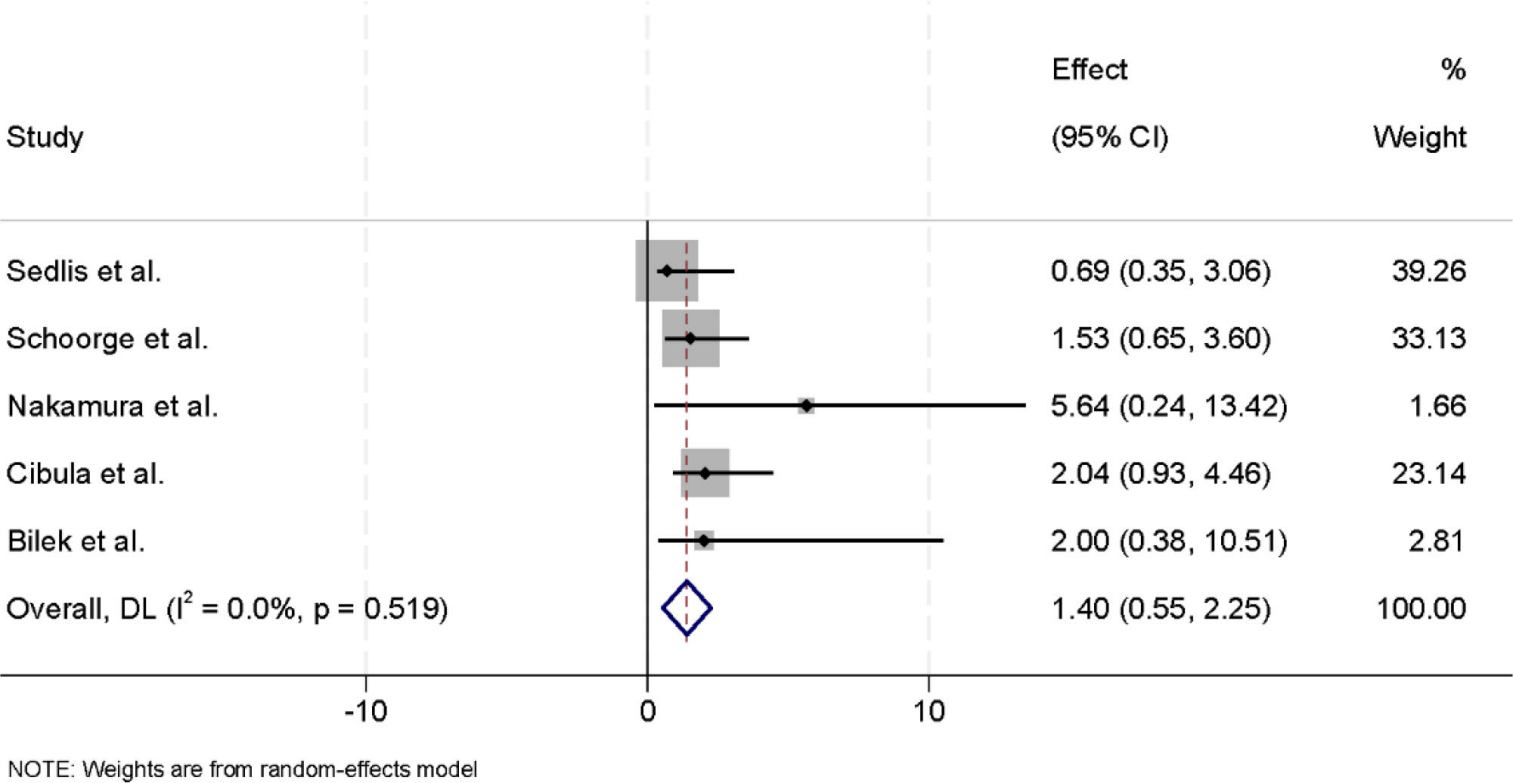
Forest plot of overall mortality comparing surgery alone with surgery plus adjuvant therapy in patients with early-stage cervical cancer. Effect sizes are presented as risk ratios (RRs) with 95% confidence intervals (CIs), and the horizontal axis is displayed on a logarithmic scale.

Five studies reported data on overall mortality among patients who underwent radical hysterectomy for early-stage cervical cancer, with or without adjuvant therapy. Statistical testing indicated no significant heterogeneity among the included studies (I² = 0.0%, p = 0.519), and therefore a fixed-effects model was applied to calculate the pooled estimate. The meta-analysis demonstrated that postoperative adjuvant therapy did not significantly influence overall mortality compared with surgery alone (RR = 1.40, 95% CI: 0.55–2.25) (**Figure 4**). The consistency of findings across studies suggests a relatively uniform treatment effect, reinforcing the reliability of the pooled estimate.

### 3.6 Subgroup analysis of recurrence outcomes

To further explore potential sources of heterogeneity in recurrence outcomes, subgroup analyses were conducted based on study design, FIGO stage, and type of adjuvant therapy. When stratified by study design, no significant difference in recurrence rates was observed between the surgery-only and adjuvant therapy groups in either randomized controlled trials (RR = 1.18, 95% CI: 0.64–2.09) or observational cohort studies (RR = 1.63, 95% CI: 0.72–2.54), suggesting that study type did not substantially influence the overall findings. Subgroup analysis by FIGO stage showed similar results. Among patients with stage IB disease, adjuvant therapy after radical hysterectomy did not significantly reduce recurrence compared with surgery alone (RR = 1.32, 95% CI: 0.61–2.14). For those with stage IIA or higher, the pooled effect remained nonsignificant (RR = 1.69, 95% CI: 0.73–3.01). These findings indicate that the benefit of adjuvant therapy was not evident across early-stage subgroups. When further stratified by type of adjuvant treatment, including radiotherapy alone, chemoradiotherapy, or multimodal regimens, no subgroup showed a statistically significant reduction in recurrence risk compared with surgery alone. The corresponding pooled effect estimates were RR = 1.44 (95% CI: 0.66–2.30) for radiotherapy and RR = 1.57 (95% CI: 0.70–2.65) for chemoradiotherapy (**Table 3**).

**Table 3:** Subgroup analysis of recurrence outcomes.

Subgroup	Model used	Pooled RR (95% CI)	I² (%)	P-value for heterogeneity	Interpretation
Overall analysis	Random-effects	1.51 (0.75–2.28)	76.2	<0.001	No significant difference
Study design					
Randomized controlled trials	Fixed-effects	1.18 (0.64–2.09)	12.4	0.312	No significant difference
Observational cohort studies	Random-effects	1.63 (0.72–2.54)	68.3	0.019	No significant difference
FIGO stage					
Stage IA–IB	Random-effects	1.32 (0.61–2.14)	71.5	0.007	No significant difference
Stage IIA	Random-effects	1.69 (0.73–3.01)	62.8	0.041	No significant difference
Type of adjuvant therapy					
Radiotherapy only	Random-effects	1.44 (0.66–2.30)	65.9	0.026	No significant difference
Chemoradiotherapy	Random-effects	1.57 (0.70–2.65)	58.1	0.078	No significant difference
Multimodal regimens (RT + CT or CRT + BT)	Random-effects	1.62 (0.68–3.10)	60.5	0.063	No significant difference

RR, risk ratio; CI, confidence interval; I², heterogeneity index; FIGO, International Federation of Gynecology and Obstetrics;

RT, radiotherapy; CT, chemotherapy; CRT, chemoradiotherapy; BT, brachytherapy.

A random-effects model was applied when I² > 50% or p < 0.10; otherwise, a fixed-effects model was used.

No subgroup demonstrated a statistically significant difference in recurrence risk between surgery alone and adjuvant therapy groups.

### 3.7 Publication bias

Publication bias was evaluated using Begg’s rank correlation test and Egger’s regression asymmetry test to assess the potential influence of small-study effects on the pooled results. The results indicated no significant evidence of publication bias among the included studies for recurrence outcomes (Begg’s test: p = 0.621; Egger’s test: p = 0.487). Similarly, for mortality outcomes, both tests suggested an absence of publication bias (Begg’s test: p = 0.707; Egger’s test: p = 0.553).

## 4 Discussion

This systematic review and meta-analysis synthesized available evidence on the oncologic impact of adjuvant therapy following radical hysterectomy in patients with early-stage cervical cancer (FIGO IA–IIA). Across eight studies comprising 1, 396 patients that reported recurrence outcomes and five studies that reported mortality, no statistically significant benefit of postoperative adjuvant therapy over surgery alone was observed. The pooled analysis showed no reduction in recurrence risk with adjuvant therapy (RR = 1.51, 95% CI 0.75–2.28), although substantial between-study heterogeneity was present (I² = 76.2%), nor was there a survival advantage in terms of overall mortality (RR = 1.40, 95% CI 0.55–2.25), with negligible heterogeneity across studies (I² = 0%). Importantly, leave-one-out sensitivity analyses confirmed the robustness of these findings, indicating that no individual study disproportionately influenced the pooled estimates. Furthermore, prespecified subgroup analyses stratified by study design (randomized versus observational), FIGO stage, and adjuvant modality (radiotherapy alone versus chemoradiotherapy) consistently failed to demonstrate a recurrence-reducing effect associated with adjuvant treatment.

These findings have direct clinical relevance, as postoperative adjuvant therapy is frequently employed after radical hysterectomy to mitigate recurrence risk in early-stage disease, particularly in the presence of adverse pathological features. However, when evaluated across unselected early-stage cohorts, our results indicate that this strategy does not translate into measurable improvements in recurrence or survival. This absence of oncologic benefit is especially noteworthy given the well-documented morbidity associated with postoperative radiotherapy and chemoradiotherapy, including genitourinary, gastrointestinal, and sexual dysfunction, which can substantially impair long-term quality of life. From a population-level and value-based care perspective, routine administration of adjuvant therapy after adequate radical surgery in early-stage cervical cancer may therefore represent overtreatment rather than therapeutic necessity. It is also notable that the included studies span several decades, encompassing both historical and more contemporary treatment eras. Although advances in surgical techniques, radiotherapy planning, and supportive care may influence absolute outcome rates, the consistency of null findings across time periods supports the biological plausibility that thorough surgical clearance alone is sufficient for disease control in a substantial proportion of early-stage cases. Collectively, these results reinforce the importance of individualized, risk-adapted postoperative management and argue against the routine use of adjuvant therapy following radical hysterectomy in unselected patients with early-stage cervical cancer.

Although substantial heterogeneity was observed in the pooled recurrence analysis, subgroup and sensitivity analyses provided useful context for interpreting the overall findings. Stratification by study design showed no significant difference in recurrence outcomes between randomized controlled trials and observational cohort studies, suggesting that methodological differences did not materially alter the direction of effect. Likewise, subgroup analyses according to FIGO stage yielded broadly consistent results, with no statistically significant recurrence reduction associated with adjuvant therapy in any early-stage subgroup examined. Further stratification by adjuvant modality, including radiotherapy alone and chemoradiotherapy, also failed to identify a subgroup with a significant recurrence benefit. These findings suggest that the absence of an apparent recurrence-reducing effect was not confined to a single study type, stage category, or treatment modality (27). Sensitivity analyses further showed that no individual study disproportionately influenced the pooled estimate. Taken together, these results support the overall stability of the main findings while also indicating that treatment intensification did not translate into a measurable oncologic advantage in the pooled early-stage population studied (14).

Recent literature provides additional context for the clinical implications of our findings. Mora-Soto et al. (14) reported that combining radical surgery with postoperative radiotherapy may substantially increase treatment-related morbidity, including urinary, gastrointestinal, sexual, and lymphatic complications, with potential long-term impairment in quality of life. In this context, our findings, which showed no significant reduction in recurrence or mortality with routine adjuvant therapy, further underscore the need to balance potential oncologic benefit against treatment-related harm. Chikazawa et al. (28) likewise reported comparable recurrence-free and overall survival between radical hysterectomy followed by adjuvant chemotherapy and primary chemoradiotherapy in selected cervical cancer populations, highlighting persistent uncertainty regarding the optimal integration of surgery and adjuvant therapy. At the same time, contemporary reviews of early-stage cervical cancer management increasingly emphasize treatment individualization and avoidance of unnecessary therapeutic intensity (13). Advances in surgical techniques, sentinel lymph node assessment, and fertility-preserving strategies aim to maintain oncologic safety while reducing morbidity. Within this evolving framework, treatment de-escalation has gained increasing attention as a means of minimizing long-term toxicity without compromising disease control (29, 30). Although much of the de-escalation literature comes from other malignancies, its central principle remains applicable to gynecologic oncology: treatment intensity should be tailored to individual risk in order to avoid overtreatment. Emerging translational research further supports precision-based approaches through improved biomarker discovery and patient stratification. Together, these developments reinforce the need for risk-adapted postoperative management in early-stage cervical cancer and are consistent with our findings that routine adjuvant therapy may not be necessary for all patients after radical hysterectomy.

An important consideration in interpreting the present findings is that the included evidence was weighted primarily toward intermediate-risk postoperative populations rather than clearly defined high-risk disease (31). Several of the core studies, including those based on Sedlis-type criteria or lymph node-negative patients with adverse pathological factors, addressed the clinical question of whether adjuvant treatment improves outcomes in intermediate-risk early-stage cervical cancer after radical hysterectomy. In this setting, our findings did not demonstrate a clear recurrence or mortality benefit associated with routine postoperative treatment. By contrast, patients with established high-risk factors, such as nodal metastasis, parametrial involvement, or positive margins, were not uniformly represented in the eligible studies and could not be reliably analyzed as an independent pooled subgroup (32). Accordingly, the conclusions of the present meta-analysis should be interpreted primarily as applying to unselected or predominantly intermediate-risk early-stage populations, rather than to guideline-defined high-risk patients, for whom postoperative chemoradiation may still remain clinically justified (33). Although the possibility of meta-regression was considered, this approach was not pursued because the review included only 8 studies, and important pathological variables, such as nodal status, margin status, parametrial involvement, LVSI, tumor size, and depth of stromal invasion, were not reported in a sufficiently uniform study-level format to support a robust adjusted analysis.

Several limitations should be acknowledged. First, substantial heterogeneity was observed in the recurrence analysis, likely reflecting differences in study design, patient selection, definitions of intermediate risk, and adjuvant treatment regimens across studies. Second, most included studies were observational, raising the possibility of confounding by indication and measurement bias. Patients receiving adjuvant therapy may have had higher baseline risk features, including larger tumor size, lymphovascular space invasion, or deeper stromal invasion, which could partly influence recurrence outcomes independent of treatment effects. Third, adjuvant regimens varied in composition and quality, and radiotherapy techniques used in older studies may not reflect contemporary conformal approaches, potentially limiting the generalizability of efficacy and toxicity outcomes. Fourth, the number of mortality events was limited, reducing statistical power to detect modest survival differences. In addition, recurrence patterns and treatment-related toxicity or quality-of-life outcomes were inconsistently reported and could not be synthesized quantitatively. Finally, although formal assessments did not indicate significant publication bias, the possibility of selective reporting cannot be entirely excluded. Future well-designed prospective studies with standardized definitions, comprehensive reporting of recurrence patterns, toxicity, and quality-of-life outcomes, and integration of clinicopathologic or biomarker-based stratification are needed to better define which patient subgroups may benefit from postoperative adjuvant therapy.

## 5 Conclusions

This meta-analysis demonstrated that adjuvant therapy after radical hysterectomy did not significantly reduce recurrence or mortality in patients with early-stage cervical cancer. Subgroup and sensitivity analyses confirmed the robustness of these findings across different study designs, FIGO stages, and treatment modalities. Routine postoperative adjuvant therapy may therefore be unnecessary for unselected early-stage cases, supporting a risk-adapted and individualized treatment strategy to minimize overtreatment and optimize long-term outcomes.

## Data Availability

The raw data supporting the conclusions of this article will be made available by the authors, without undue reservation.
