# Supplementary material for: Impact of adjuvant therapy after radical hysterectomy for early-stage cervical cancer on mortality and recurrence: a systematic review and meta-analysis
Source: Front Oncol. 2026 Apr 29;16:1743116. doi: 10.3389/fonc.2026.1743116 (PMC13167952; doi:10.3389/fonc.2026.1743116)
Supplement: Supplementary file 1 [file Table1.docx]

Supplementary Table 1. Detailed Search Strategies for Each Database

| Database | Search Strategy |
| --- | --- |
| PubMed | ((“Uterine Cervical Neoplasms”[MeSH] OR “Cervical Cancer” OR “Cervical Carcinoma” OR “Cervical Neoplasm*” OR “Cancer of the Cervix” OR “Cervix Cancer”) AND (“Hysterectomy, Radical”[MeSH] OR “Radical Hysterectomy” OR “Extended Hysterectomy” OR “Wertheim Hysterectomy” OR “Surgical Treatment” OR Surgery) AND (“Adjuvant Therapy”[MeSH] OR “Adjuvant Treatment” OR “Postoperative Therapy” OR “Postoperative Radiotherapy” OR “Adjuvant Radiotherapy” OR “Adjuvant Chemotherapy” OR “Concurrent Chemoradiotherapy” OR “Combined Modality Therapy”) AND (“Mortality”[MeSH] OR “Survival”[MeSH] OR “Overall Survival” OR “Disease-Free Survival” OR “Progression-Free Survival” OR “Recurrence” OR “Relapse” OR “Treatment Outcome” OR “Prognosis”)) |
| Embase | (‘uterine cervical cancer’/exp OR ‘cervical cancer’:ab,ti OR ‘cervical carcinoma’:ab,ti OR ‘cervical neoplasm*’:ab,ti OR ‘cancer of the cervix’:ab,ti) AND (‘radical hysterectomy’/exp OR ‘radical hysterectomy’:ab,ti OR ‘extended hysterectomy’:ab,ti OR ‘wertheim hysterectomy’:ab,ti OR surgery:ab,ti) AND (‘adjuvant therapy’/exp OR ‘adjuvant treatment’:ab,ti OR ‘postoperative therapy’:ab,ti OR ‘adjuvant radiotherapy’:ab,ti OR ‘adjuvant chemotherapy’:ab,ti OR ‘concurrent chemoradiotherapy’:ab,ti OR ‘combined modality therapy’:ab,ti) AND (‘mortality’/exp OR survival:ab,ti OR ‘overall survival’:ab,ti OR ‘disease free survival’:ab,ti OR recurrence:ab,ti OR relapse:ab,ti OR prognosis:ab,ti) |
| Web of Science | TS=(“cervical cancer” OR “cervical carcinoma” OR “uterine cervical neoplasm*” OR “cancer of the cervix” OR “cervix cancer”) AND TS=(“radical hysterectomy” OR “extended hysterectomy” OR “wertheim hysterectomy” OR “surgical treatment” OR surgery) AND TS=(“adjuvant therapy” OR “adjuvant treatment” OR “postoperative therapy” OR “adjuvant radiotherapy” OR “adjuvant chemotherapy” OR “concurrent chemoradiotherapy” OR “combined modality therapy”) AND TS=(“mortality” OR “overall survival” OR “disease-free survival” OR “progression-free survival” OR “recurrence” OR “relapse” OR “prognosis”) |
| Cochrane Library | (“Uterine Cervical Neoplasms” OR “cervical cancer” OR “cervical carcinoma” OR “cervical neoplasm*” OR “cancer of the cervix”) in Title Abstract Keyword AND (“radical hysterectomy” OR “extended hysterectomy” OR “wertheim hysterectomy” OR “surgical treatment” OR surgery) in Title Abstract Keyword AND (“adjuvant therapy” OR “adjuvant treatment” OR “postoperative therapy” OR “adjuvant radiotherapy” OR “adjuvant chemotherapy” OR “concurrent chemoradiotherapy” OR “combined modality therapy”) in Title Abstract Keyword AND (“mortality” OR “overall survival” OR “disease-free survival” OR “progression-free survival” OR “recurrence” OR “relapse” OR “prognosis”) |

Notes:

The search period covered from database inception to October 1, 2025.

No language restrictions were applied; non-English studies with English abstracts were included.

The Boolean operators AND and OR were used to combine search terms appropriately.

The search strategies were adapted to match the specific syntax and controlled vocabulary (e.g., MeSH in PubMed, Emtree in Embase) of each database.
